# Supplementary material for: Holistic vibrational spectromics assessment of human cartilage for osteoarthritis diagnosis
Source: Biomed Opt Express. 2024 Jun 13;15(7):4264–80. doi: 10.1364/BOE.520171 (PMC11249685; doi:10.1364/BOE.520171)
Supplement: Supplementary file 1 [file boe-15-7-4264-s001.pdf]

Supplemental Document

Biomedical Optics EXPRESS

Holistic vibrational spectromics assessment of human cartilage for osteoarthritis diagnosis: supplement

HIROKI COOK,[1,2] ANNA CRISFORD,[1,2,3] KONSTANTINOS BOURDAKOS,[1,2] DOUGLAS DUNLOP,[4] RICHARD O. C. OREFFO,[2,3] AND SUMEET MAHAJAN[1,2,5,*]

[1] *School of Chemistry, Faculty of Engineering and Physical Sciences, University of Southampton, Southampton SO17 1BJ, UK*
[2] *Institute for Life Sciences, University of Southampton, Southampton SO17 1BJ, UK*
[3] *Human Development Health, Faculty of Medicine, Southampton SO16 6YD, UK*
[4] *University Hospital Southampton NHS Foundation Trust, Southampton, SO16 6YD, UK*
[5] *Department of Biotechnology, Inland Norway University of Applied Sciences, N-2317 Hamar, Norway*
[*] *S.Mahajan@soton.ac.uk*

This supplement published with Optica Publishing Group on 13 June 2024 by The Authors under the terms of the Creative Commons Attribution 4.0 License in the format provided by the authors and unedited. Further distribution of this work must maintain attribution to the author(s) and the published article's title, journal citation, and DOI.

Supplement DOI: https://doi.org/10.6084/m9.figshare.25943884

Parent Article DOI: https://doi.org/10.1364/BOE.520171

Holistic Vibrational Spectromics Assessment of Human Cartilage for Osteoarthritis Diagnosis

HIROKI COOK,[1,2] ANNA CRISFORD,[1,2,3] KONSTANTINOS BOURDAKOS,[1,2] DOUGLAS DUNLOP,[4] RICHARD OC OREFFO,[2,3] AND SUMEET MAHAJAN [1,2,5*]

[1]*School of Chemistry, Faculty of Engineering and Physical Sciences, University of Southampton, Southampton, SO17 1BJ, UK*
[2]*Institute for Life Sciences, University of Southampton, Southampton, SO17 1BJ, UK*
[3]*Human Development Health, Faculty of Medicine, Southampton, SO16 6YD, UK*
[4]*University Hospital Southampton NHS Foundation Trust, Southampton, SO16 6YD, UK*
[5]*Department of Biotechnology, Inland Norway University of Applied Sciences, N-2317 Hamar, Norway*

SUPPLEMENTAL DOCUMENT

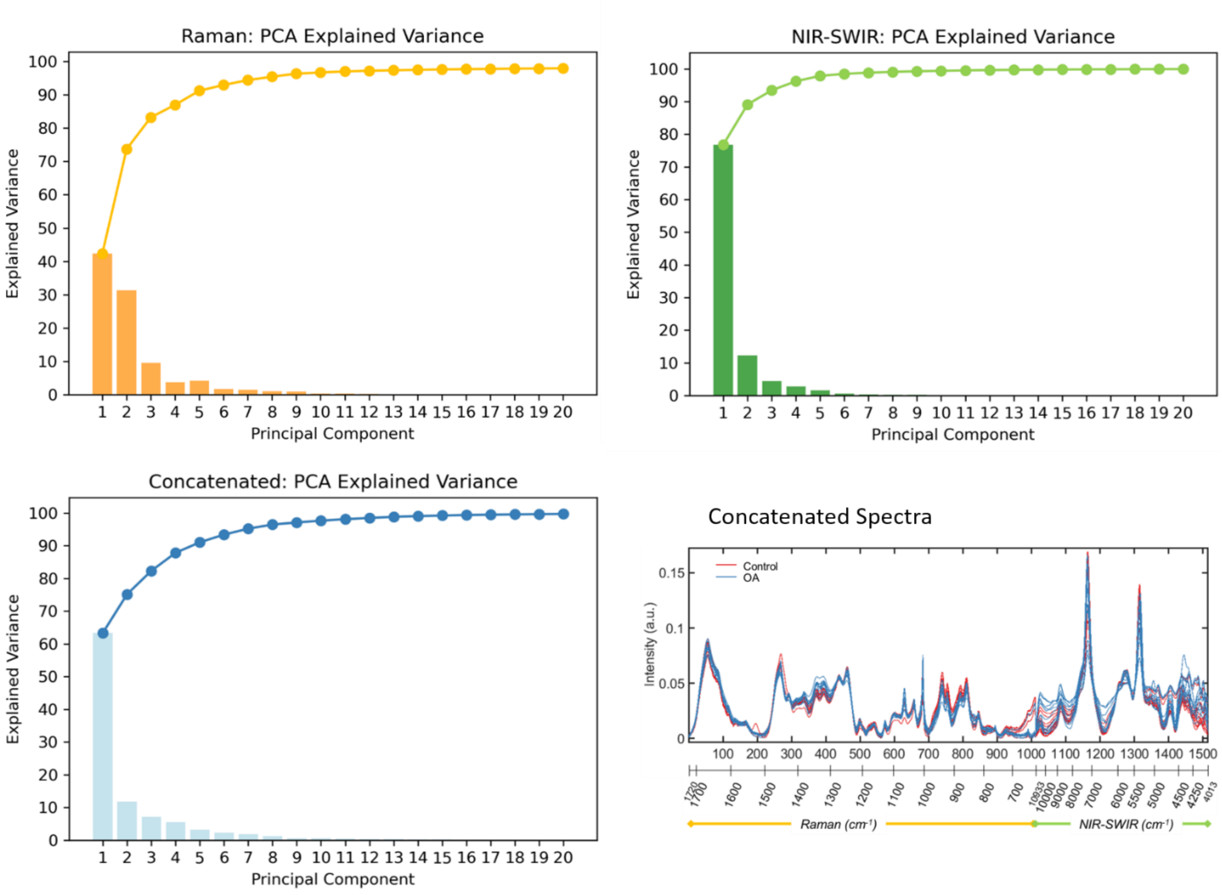

Fig. S1. Cumulative Explained Variance for increasing number of Principal Components considered for each spectroscopic modality, Raman, NIR-SWIR and Concatenated. Bottom Right: Class means for concatenated spectra from all patients, with Raman (614 – 1722 cm^{-1}) and NIR-SWIR (11,127 – 3993 cm^{-1}) regimes demarcated in abstracted Spectromics fingerprint.

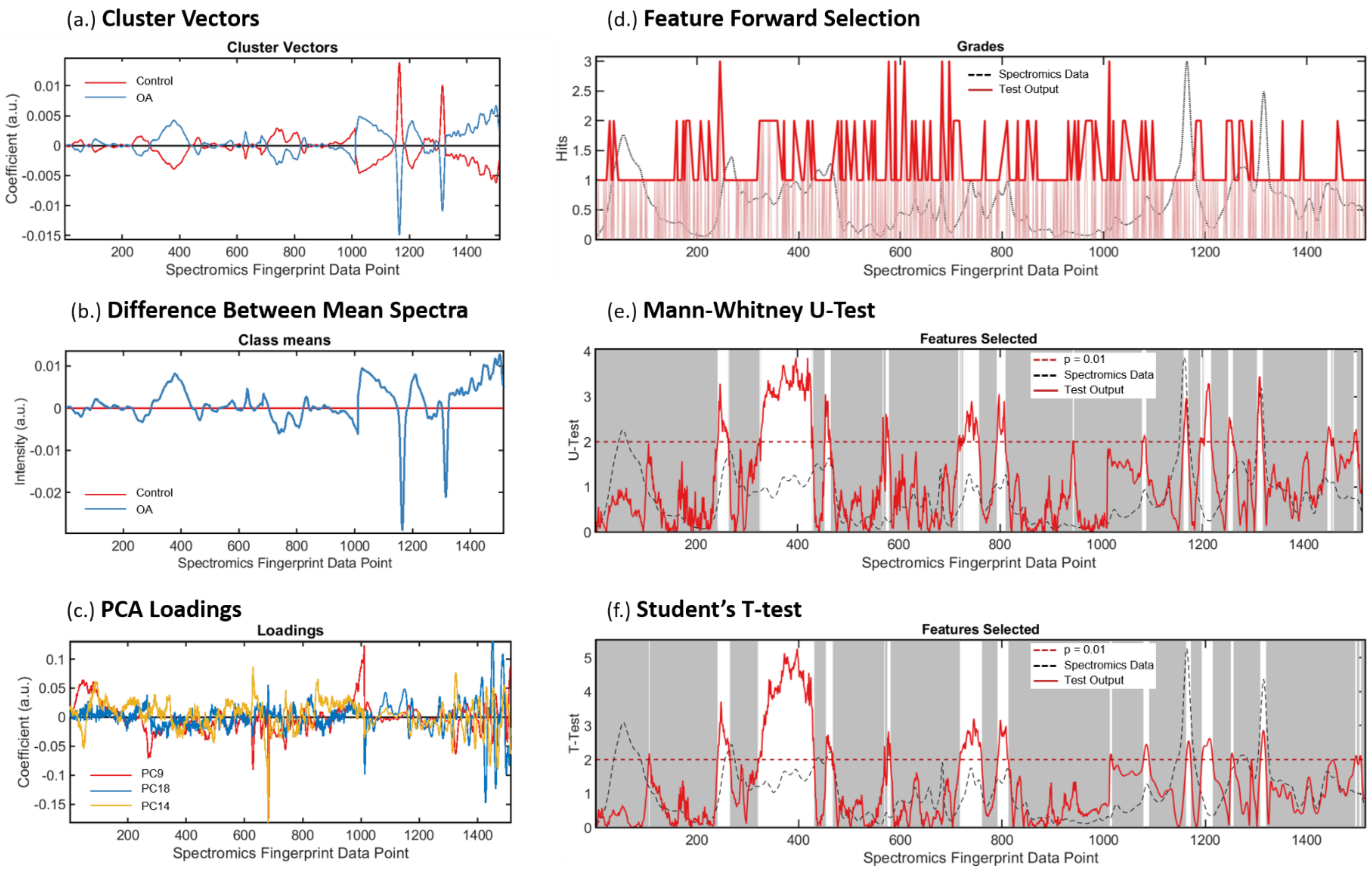

Fig. S2. Feature selection to identify spectral biomarkers for articular cartilage diagnostics. Results were corroborated between each independent statistical analysis to highlight wavenumbers most contributing to tissue classification. (a.) Cluster Vector Analysis for class clustering, (b.) Difference Between Mean Control and OA Spectra, (c.) PCA-LDA loadings scores, (d.) Feature Forward Selection, (e.) Mann-Whitney U-Test, (f.) Student's T-Test

Supplementary Table 1: Spectral Biomarkers identified by corroboration of independent statistical tests to identify osteoarthritis vs control class discriminating peaks. Features in agreement under 3 and 4 tests (no. of hits) are displayed alongside the corresponding spectral position and attributed chemical vibration. [1,2] [1,3] [4]

Hits	Spectromics Data Point	Wavenumber (cm^{-1})	Modality	1st Derivative Attributed Peak	2nd Derivative Attributed Peak	Assignment
**4***	373 – 381	1342.64 – 1334.18	Raman	-	-	CH, GAGs at 1342 cm^{-1}
**4***	733 – 738	946.56 – 940.82	Raman	-	-	C–C deformation of aggrecan / C–O–C stretching of GAGs at 937 – 941 cm^{-1}
**4***	806 – 808	862.16 – 859.83	Raman	-	-	C–C Stretching, Proline, Collagen at 856 – 859 cm^{-1}
**4***	1011 / 1015	617.056 / 10819.9	Raman / NIR-SWIR	-	-	Spectromics Artefact: Raman to NIR-SWIR transition
**4***	1164, 1168	7158.55, 7094.38	NIR-SWIR	7174.78	7062.73	O-H Stretching (1st Overtone) at 7280 - 6040, 7460 - 6780 cm^{-1}
**4***	1207 – 1211	6524.79 – 6471.57	NIR-SWIR	-	6254.84	N-H stretch (-CONH, 1st overtone) at 6352 cm^{-1}
**4***	1313 – 1316	5356.95 – 5333.09	NIR-SWIR	5342.02	5280.17	Bound & Free water at 5200 cm^{-1}
**4***	1443 – 1449	4402.48 – 4366.62	NIR-SWIR	4408.17, 4420.64	4372.55, 4384.47	C–H bend (protein, 2nd overtone), at 4350 cm^{-1}
**4***	1500 – 1503	4084.29 – 4068.83	NIR-SWIR	4073.97, 4058.60	4068.83, 4073.97	No precedent
**3***	106, 107	1616.69, 1615.69	Raman	-	-	Amide I at 1612–1696 cm^{-1}
**3***	245 – 247	1475.99 –1473.94	Raman	-	-	CH_2 deformation/scissoring; protein & lipids at 1441 – 1460 cm^{-1}
**3***	264 – 269	1456.43 – 1451.27	Raman	-	-	CH_2/CH_3 scissoring; collagen & other protein at 1451 cm^{-1}
**3***	455 – 463	1255.22 – 1246.61	Raman	-	-	C-N stretching (Amide III) at 1230 – 1280 cm^{-1}
**3***	575 – 577	1124.38 – 1122.17	Raman	-	-	Pyranose ring at 1127 – 1163 cm^{-1}
**3***	629, 630	1064.33, 1063.21	Raman	-	-	SO_3^- stretching in sulphated GAGs, PGs at 1060 – 1064 cm^{-1}
**3***	1083, 1084	8767.51, 8744.18	NIR-SWIR	8719.98	8577.59	C–H stretching (2nd overtone) at 8820 – 8060, 8695 - 8197 cm^{-1}

References

1. L. Rieppo, J. Töyräs, and S. Saarakkala, "Vibrational spectroscopy of articular cartilage," Appl. Spectrosc. Rev. **52**(3), 249–266 (2017).

2. I. O. Afara and A. Oloyede, "Resolving the Near-Infrared Spectrum of Articular Cartilage," Cartilage **13**(1_suppl), 729S-737S (2021).

3. P. Casal-Beiroa, V. Balboa-Barreiro, N. Oreiro, S. Pértega-Díaz, F. J. Blanco, and J. Magalhães, "Optical biomarkers for the diagnosis of osteoarthritis through raman spectroscopy: Radiological and biochemical validation using ex vivo human cartilage samples," Diagnostics **11**(3), (2021).

4. R. Kumar, G. Singh, K. Grønhaug, N. Afseth, C. de Lange Davies, J. Drogset, and M. Lilledahl, "Single Cell Confocal Raman Spectroscopy of Human Osteoarthritic Chondrocytes: A Preliminary Study," Int. J. Mol. Sci. **16**(12), 9341–9353 (2015).
